# 

**Published:** 1819-07

**Authors:** 


### TO CORRESPONDENTS, &c.

*The Communications of* Doctors Kinglake, Painchaud, *and* Von Embden; *and* Surgeons Overend, Sutliffe, *and* Down; *and* Senex; *will appear in the next Number.*

*The Meteorological Table, and the List of Books, are necessarily deferred until the ensuing Number.*

